# Sensorineural Hearing Loss as a Complication of COVID-19 and the COVID-19 Vaccine

**DOI:** 10.7759/cureus.47582

**Published:** 2023-10-24

**Authors:** Zev Hirt, Avraham Kohanzadeh, Marc Gibber

**Affiliations:** 1 Otorhinolaryngology - Head and Neck Surgery, Albert Einstein College of Medicine, Bronx, USA; 2 Otorhinolaryngology - Head and Neck Surgery, Montefiore Medical Center, Wakefield Campus, Bronx, USA

**Keywords:** otorhinolaryngology, covid-19 vaccine, snhl, covid 19, hearing loss

## Abstract

The relationship between COVID-19 and sensorineural hearing loss (SNHL) continues to solidify in light of a growing body of evidence. In addition to the well-established COVID-19 symptoms and sequelae, otolaryngologists have observed an increased incidence of SNHL in patients with COVID-19 and following COVID-19 immunizations. Although the precise mechanisms behind this association remain unclear, various hypotheses are discussed within the scientific literature. This case report expands on the relationship between COVID-19 and SNHL by discussing a unique case of bilateral tinnitus and subsequent SNHL shortly following COVID-19 immunization. It contributes to the growing body of evidence associating COVID-19 with SNHL, underlining the importance of further research to understand potential causal mechanisms. It underscores the clinical importance of monitoring hearing in COVID-19 patients and those receiving immunizations, advocating for increased awareness among clinicians to facilitate early identification and appropriate intervention in cases of COVID-19-related hearing loss.

## Introduction

Background

In the wake of the COVID-19 pandemic, reports of various patient presentations with the novel coronavirus have become commonplace. Sensory deficits such as anosmia and hypogeusia are some of the most well-known, yet others are still being uncovered. It has been reported that up to 30% of COVID-19 cases have a neurological manifestation, but the scientific literature is still in its early stages [1].

In addition to the more well-known manifestations of COVID-19, otolaryngologists have noticed an increase in the rate of new sensorineural hearing loss (SNHL) cases in the context of a COVID-19 diagnosis and COVID-19 vaccine administration. The exact nature of this relationship remains largely unknown. 

Hearing loss is the most common sensory deficit globally. In 2023, the World Health Organization reported that more than 1.5 billion people live with hearing loss. Population studies on the prevalence of hearing loss in the U.S. indicate that it affects approximately 23% of individuals aged 12 or older. Furthermore, older adults experience a higher prevalence and more severe forms of hearing loss compared to younger adults [2]. Tracking the incidence of a chronic disease like hearing loss can be challenging, but less so for sudden sensorineural hearing loss, defined as the loss of 30 dB or greater over at least three sequential audiometric frequencies over a 72-hour span. 

Common risk factors for SNHL include presbycusis, noise exposure, infections, ototoxic medications, and idiopathic causes. In regard to treatment, cochlear implants and hearing aids are the standard of care.

Methods

In this study, we conducted a large-scale chart review of COVID-19 cases between July 2020 to January 2023 in the Montefiore healthcare system. With the help of Atlas, an open-source technology that allows the user to perform data analytics with patient data, we were able to parse through patient records to find the relevant data for this research topic. Connecting the Atlas system with the Epic EMR system, data analytics were possible for all patients in Bronx-Montefiore sites that use Epic. Atlas was used to help isolate 234 cases of Covid-19 that were subsequently diagnosed with SNHL for the first time, within approximately one year of a Covid-19 positive test between July 2020 and July 2021. Further chart reviews were conducted for patients after July 2021 without the use of Atlas.

This case report will discuss the experience of an individual who was diagnosed with hearing loss in the aftermath of a COVID-19 vaccination.

## Case presentation

A 68-year-old female with a past medical history of hypertension and hyperlipidemia presented to urgent care for the gradual onset of ringing in her ears. Four weeks earlier, she was in her usual state of health when she received her third dose of the COVID-19 Pfizer Monovalent vaccine. Within four days, she developed bilateral tinnitus of low to moderate volume, which had persisted since that time. She had no significant history of noise exposure, no specific otologic history, no hearing fluctuations, no current ear pain, no ear drainage, and no vertigo symptoms. She felt that she had good hearing in both ears and had not noted a change in her hearing associated with the tinnitus. Her medications include famotidine, felodipine, latanoprost, losartan, and pravastatin. She was referred to schedule a follow-up appointment with an ENT specialist in an outpatient setting. 

At a telemedicine visit with her internist two weeks later, the patient complained of continuing symptoms of tinnitus. 

**Figure 1:**
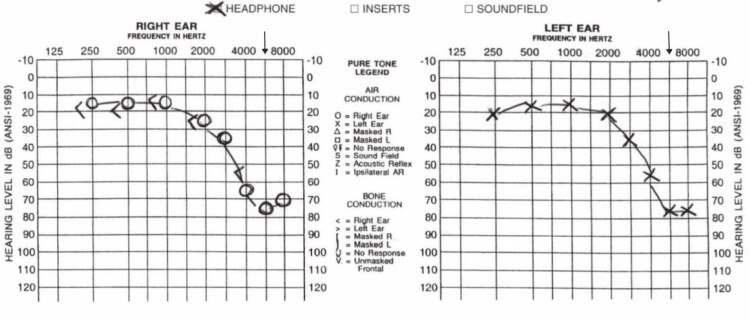
Initial audiology report. The patient had bilateral symmetric normal through 2000 Hz dropping to severe sensorineural hearing loss (SNHL) by 6000 Hz, consistent with SNHL. Tympanograms were within normal limits (WNL). Arrows point to 6000 hz in the right and left ear.

Four months after her original presentation, the patient visited an ENT in an ambulatory setting. A Tinnitus Handicap Inventory (THI) was taken, and she received a score of 24, indicating a mild handicap. She also received audio tracings (using pure tone audiometry) and was found to have bilateral symmetric normal through 2000 Hz dropping to severe SNHL by 6000 Hz, concerning for SNHL (Figure 1). Her speech recognition ability was excellent: word recognition score (WRS) was 100% auris utraque (AU). Tympanograms were within normal limits. No prior audiometric evaluations were available, and no relevant imaging was available. She was not given any treatment and was advised to follow up in 6 months, but she never did.

## Discussion

Both COVID-19 and the COVID-19 vaccine have been reported in the literature to be associated with a new onset of SNHL [3,4]. However, SNHL’s multifactorial etiology has left researchers challenged to describe a precise pathophysiology to connect the two. Viral infection is known as a common risk factor for SNHL, and there are many hypotheses about how a virus like SARS-CoV-2 could lead to SNHL. By April 2020, only a couple of months into the COVID-19 pandemic, Sriwijitalai et al. had described a connection between COVID-19 and SNHL [3]. Some hypothesize that because the inner ear is susceptible to viruses, it could have a direct pathophysiology. Research has shown that the SARS-CoV-2 virus could access the inner ear through the olfactory nerve. The olfactory nerve has been implicated in symptoms of anosmia in COVID-19 patients and may play a role here as well [5]. ACE2 inhibitors have also been shown to be expressed in the inner ear, along with the transmembrane protease serine 2 (TMPRSS2) and FURIN cofactors which allow for viral entry [6].

Though the viral infection itself seems to have some connection to SNHL, it is also possible that treatments for coronavirus and other comorbidities are responsible for the increased incidence of hearing loss. For example, in the early stages of the COVID-19 pandemic, chloroquine and hydroxychloroquine were considered as possible treatments but have been shown to have ototoxic risks [7]. Additionally, azithromycin, lopinavir-ritonavir, interferon, ribavirin, and ivermectin have exhibited ototoxic side effects, possibly leading to SNHL in patients suffering from COVID-19 [8].

In June 2022, Fancello et al. reviewed 63 new cases of SNHL following COVID-19 in the literature up to that point, highlighting that 19% of them were associated with ototoxic treatments [9]. Additionally, 87.3% of the SNHL cases were associated with other symptoms, and 25% occurred with another chemosensory deficit (anosmia, hyposmia, ageusia, and dysgeusia) [9].

During that same month, Pisani et al. published a study considering a relationship between the COVID-19 vaccine and SNHL [10]. They hypothesized that there could be rare cases of inner ear damage resulting in sudden SNHL, tinnitus, dizziness vertigo, or a combination of these symptoms [10]. However, there is yet to be a definitive study showing a relationship between the two.

## Conclusions

This case study adds to the growing body of evidence suggesting a possible association between COVID-19 and SNHL. Further research is needed to elucidate the exact mechanisms and establish a definitive link. The case underscores the importance of monitoring and evaluating hearing function in individuals with COVID-19, as well as the need for ongoing surveillance and management of patients who receive the COVID-19 vaccine. Increased awareness among healthcare professionals can lead to early identification and appropriate interventions for COVID-19-related hearing loss.

## References

[1] Ahmad I, Rathore FA Neurological manifestations and complications of COVID- 19: a literature review. J Clin Neurosci2020.

[2] Goman AM, Lin FR (2016). Prevalence of hearing loss by severity in the United States. Am J Public Health.

[3] Sriwijitalai W, Wiwanitkit V (2020). Hearing loss and COVID-19: a note. Am J Otolaryngol.

[4] Jeong J, Choi HS (2021). Sudden sensorineural hearing loss after COVID-19 vaccination. Int J Infect Dis.

[5] Najafloo R, Majidi J, Asghari A (2021). Mechanism of anosmia caused by symptoms of COVID-19 and emerging treatments. ACS Chem Neurosci.

[6] Jeong M, Ocwieja KE, Han D (2021). Direct SARS-CoV-2 infection of the human inner ear may underlie COVID-19-associated audiovestibular dysfunction. Commun Med (Lond).

[7] Prayuenyong P, Kasbekar AV, Baguley DM (2020). Clinical Implications of chloroquine and hydroxychloroquine ototoxicity for COVID-19 treatment: a mini-review. Front Public Health.

[8] Little C, Cosetti MK (2021). A narrative review of pharmacologic treatments for COVID-19: safety considerations and ototoxicity. Laryngoscope.

[9] Fancello V, Fancello G, Hatzopoulos S, Bianchini C, Stomeo F, Pelucchi S, Ciorba A (2022). Sensorineural hearing loss post-COVID-19 infection: an update. Audiol Res.

[10] Pisani D, Gioacchini FM, Viola P (2022). Audiovestibular disorders after COVID-19 vaccine: is there an association?. Audiol Res.

